# Deep Learning Post-Filtering Using Multi-Head Attention and Multiresolution Feature Fusion for Image and Intra-Video Quality Enhancement

**DOI:** 10.3390/s22041353

**Published:** 2022-02-10

**Authors:** Ionut Schiopu, Adrian Munteanu

**Affiliations:** Department of Electronics and Informatics, Vrije Universiteit Brussel, Pleinlaan 2, 1050 Brussels, Belgium; acmuntea@etrovub.be

**Keywords:** deep learning, post-filtering, quality enhancement, image compression, video coding

## Abstract

The paper proposes a novel post-filtering method based on convolutional neural networks (CNNs) for quality enhancement of RGB/grayscale images and video sequences. The lossy images are encoded using common image codecs, such as JPEG and JPEG2000. The video sequences are encoded using previous and ongoing video coding standards, high-efficiency video coding (HEVC) and versatile video coding (VVC), respectively. A novel deep neural network architecture is proposed to estimate fine refinement details for full-, half-, and quarter-patch resolutions. The proposed architecture is built using a set of efficient processing blocks designed based on the following concepts: (i) the multi-head attention mechanism for refining the feature maps, (ii) the weight sharing concept for reducing the network complexity, and (iii) novel block designs of layer structures for multiresolution feature fusion. The proposed method provides substantial performance improvements compared with both common image codecs and video coding standards. Experimental results on high-resolution images and standard video sequences show that the proposed post-filtering method provides average BD-rate savings of 31.44% over JPEG and 54.61% over HEVC (x265) for RGB images, Y-BD-rate savings of 26.21% over JPEG and 15.28% over VVC (VTM) for grayscale images, and 15.47% over HEVC and 14.66% over VVC for video sequences.

## 1. Introduction

In image compression, the main objective is to develop efficient algorithms that minimize the amount of data required to represent the visual information. Nowadays, the consumption of image and video content is constantly increasing which calls for the design of novel compression methods with a highly increased coding performance.

Current compression methods employed on images include conventional image codecs such as JPEG [1] and JPEG2000 [2], denoted here simply J2K. The JPEG codec [1] was developed by the Joint Photographic Experts Group (JPEG), being the most common format for image compression. The J2K standard [2] improves the compression performance over its predecessor and offers several functionalities including resolution and quality scalability and region of interest coding. JPEG and J2K lossy compressed images often suffer from compression artifacts such as blocking artifacts, color bleeding, and ringing effects. JPEG is designed based on the discrete cosine transform (DCT), the block-based nature of this codec being associated with blocking effects at high compression ratios. The global nature of the discrete wavelet transform (DWT) employed in J2K overcomes the blocking effects typical for JPEG compression, but ringing effects and color bleeding are observed at high compression ratios.

A common solution to enhance the quality of lossy images is to employ different filtering techniques which reduce the effect of coding artifacts. In this work, we propose a novel filtering method based on convolutional neural networks (CNNs) designed to enhance the quality of high-resolution images and video sequences by post-processing the decoded images and video frames without applying other modification to the corresponding coding framework.

In our prior work, we propose to replace the built-in filtering module in HEVC [3] with efficient deep learning (DL)-based filtering tools and enhance the quality of HEVC-compressed video and light field (LF) images. In [4], we propose a frame-wise multiscale CNN for quality enhancement of HEVC decoded videos, built based on inception and residual learning blocks (IResLBs) [5], where additional information is extracted from the HEVC decoder to guide the network. In [6], we propose a multiscale CNN to enhance the quality of LF images using macro-pixel volumes. In this work, we introduce a novel DL-based filtering method for enhancing the quality of high-resolution lossy compressed RGB/grayscale images and video sequences. The proposed filtering method follows a simple post-processing approach where a neural network is employed to estimate refinement details which are subsequently added to the decoded images/frames in order to enhance their quality.

In summary, the novel contributions and findings of this paper are as follows: **(1)** an efficient CNN-based post-filtering method is proposed, where the architecture is designed based on the following concepts: *(i)* the multi-head attention mechanism for refining the feature maps, *(ii)* the weight sharing concept for reducing the network complexity, and *(iii)* novel block designs of layer structures for multiresolution feature fusion; **(2)** the current resolution patch is processed using feature maps extracted from both input patch and higher resolution patches; **(3)** the network is trained to estimate the refinement details at full-, half-, and quarter-patch resolutions; **(4)** the complex experimental evaluation over RGB/grayscale images and video sequences demonstrate that the proposed method offers substantial performance improvements when filtering images encoded by common lossy codecs and video sequences encoded by recent video coding standards, by reducing the coding artifacts specific to each codec.

The remainder of this paper is organized as follows. Section 2 overviews the state-of-the-art methods for image and video quality enhancement. Section 3 describes the proposed filtering method. Section 4 presents the experimental validation on RGB images, grayscale images, and video sequences. Finally, Section 5 draws the conclusions of this work.

## 2. Related Work

In recent years, machine learning (ML) techniques rapidly gained popularity and novel DL-based tools were proposed to replace, in conventional frameworks, specific coding modules such as transform [7], prediction [5,8,9,10], rate control [11], and interpolation [12], to name a few.

The design of novel DL-based tools to integrate into HEVC became a hot research topic, and filtering tools were proposed. In [13], an artifact reduction CNN (AR-CNN) architecture is proposed to reduce the artifacts in the JPEG compressed images using a sequence of four convolution layers. ARCNN was one of the first architectures that was able to achieve more than 1 dB improvement in PSNR over the JPEG compression on classical test images. In [14], the authors proposed a super-resolution CNN (SRCNN) to obtain high-resolution images from the corresponding low-resolution images in an end-to-end manner that outperforms the state-of-the-art in both reconstruction quality and computational speed. In [15], the authors proposed the variable-filter-size residue-learning CNN (VRCNN) architecture built based on ARCNN [13] and employed to replace the conventional HEVC built-in filters, de-blocking filter (DBF) [16] and sample adaptive offset (SAO) [17], when filtering the HEVC reconstructed intra-frames. In [18], a VRCNN architecture with batch normalization is proposed, where the new method is called VRCNN-BN and provides competitive results when enhancing the quality of HEVC decoded sequences. An iterative post-filtering method based on an RNN design was proposed in [19]. In [20], the authors employ a CNN-based autoencoder to estimate the radial blur and enhance the deblurred image. In [21], an image enhancement algorithm based on image-derived graph for weak illumination images is proposed. In [22], a DL-based hue-correction scheme is proposed based on the constant-hue plane in the RGB color space for image enhancement. In [23], a post-filtering DL-based method for quality enhancement of brain images is proposed, where the method operates on 3D volumetric data with a high dynamic range. In [24], a dual-stream recursive residual network is proposed, which consists of structure and texture streams for separately reducing the specific artifacts related to high-frequency or low-frequency components in JPEG compressed images. The method is called STRNN and it is the current state-of-the-art method for artifact reduction of JPEG compressed grayscale images. In [25], a novel multilevel progressive refinement generative adversarial network (MPRGAN) is employed to filter the intra-coded frames, where the generative network refines the reconstructed frame progressively in order to maximize the error of the adversarial network when distinguishing between the enhanced frame and the original frame. In [26], the authors proposed the frame enhancement CNN (FECNN) architecture which contains nine convolutional layers and introduces the residual learning scheme for filtering intra-frames to achieve a fast convergence speed in network training and also a higher filtering performance compared with VRCNN. In [27], a residual-based video restoration network (residual-VRN) is proposed based on residual learning blocks to enhance the quality of decoded HEVC intra-frames. In [28], a recursive residual CNN (RRCNN) architecture is proposed based on the recursive learning and residual learning schemes, which provides an important advantage over the state-of-the-art methods.

In one approach, DL-based filtering tools are introduced inside the main coding loop to filter the inter-predicted frames. In [29], based on the SRCNN [14] structure, the authors proposed a CNN architecture for in-loop filtering called IFCNN. IFCNN was designed to replace the HEVC built-in SAO [17] filter and to perform frame filtering after DBF [16] in the HEVC framework. In [30], the authors proposed a deep CNN architecture called DCAD employed to perform quality enhancement for all intra- and inter-frames after applying the traditional HEVC built-in DBF [16] and SAO filters [17]. In [31], the authors proposed a CNN architecture called RHCNN which contains residual highway units designed based on residual learning units to enhance the HEVC reconstructed frames. In [32], the authors proposed to perform content-aware CNN-based filtering of the HEVC decoded frames. In [33], a partition-aware architecture is proposed, where the CU partition size is used as additional information. In [34], a GAN-based perceptual training strategy is employed to post-process VVC and AV1 results. In [35], a novel architecture which exploits multilevel feature review residual dense blocks is proposed.

In another approach, DL-based filtering tools were proposed to filter the current inter-coded frame by making use of multiple previously reconstructed frames. In [36], the authors proposed a DL-based multi-frame in-loop filter (MIF) to enhance the current frame using several previously reconstructed frames. In [37], a multi-channel long-short-term dependency residual network (MLSDRN) was proposed to perform quality enhancement in addition to the DBF&SAO filtering. In [38], the authors proposed a filtering method based on the Decoder-side scalable convolutional neural network (DS-CNN), where a DS-CNN-I model is employed to enhance the intra-coded frames. In [39], the authors proposed a DL-based filtering method, QENet, designed to work outside the HEVC coding loop. However, the QENet still makes use of multiple frames considering the temporal correlations among them when filtering the inter-coded frames. In summary, in recent years, several deep-learning-based solutions were proposed to either replace the built-in HEVC in-loop filtering methods or to further refine the reconstructed frame. This is in contrast to the recent post-filtering deep-learning-based methods which prove that an improve coding solution is ordained by simply removing the built-in HEVC in-loop filtering methods.

The research area of neural-network-based loop filtering is intensively studied at the MPEG meetings. Many contributions [40,41,42,43] were recently proposed to enhance the VVC quality. In recent years, the attention mechanism [44] has become a powerful tool for improving the network performance. In [45], the attention module is used for low-light image enhancement. In [46], a new non-local attention module is proposed for low-light image enhancement using multiple exposure image sequences.

In this paper, we follow an approach where no modifications are applied to the codec, where the proposed CNN-based post-filtering method is employed to further filter the reconstructed image or decoded video frame. The quality enhancement experiments are carried out on three types of data: RGB images, grayscale images, and intra-predicted video frames. The proposed filtering method enhances the quality of lossy images encoded using traditional image codes, such as JPEG [1] and J2K [2], and of video sequences encoded using the latest video coding standards, HEVC [3] and VVC [47].

## 3. Proposed Method

**Figure 1:**
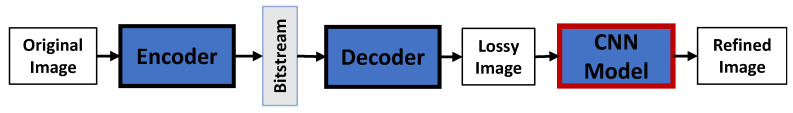
Flowchart of the proposed post-filtering approach.

Figure 1 depicts the proposed CNN-based filtering method which is designed to simply post-process a lossy image compressed using a traditional image or video codec. One can note that no modifications are applied to the encoder–decoder framework. The input image/frame is split into h×w blocks which are used as input patches for the deep neural network.

**Figure 2:**
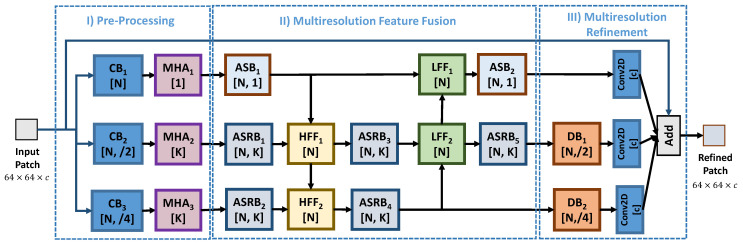
Proposed attention-based shared weights quality enhancement convolutional neural network, which is built using the following blocks: convolutional Block (CB), deconvolutional block (DB), attention-based shared weights block (ASB), attention-based shared weights residual block (ASRB), multi-head attention block (MHA), low-resolution feature fusion (LFF), and high-resolution feature fusion (HFF). The layer structure of each building block is presented in Figure 3.

Figure 2 depicts the proposed network architecture called attention-based shared weights quality enhancement convolutional neural network (ASQE-CNN). ASQE-CNN is designed based on the following three important concepts: *(i)* the attention mechanism, where channel and spatial attention are employed to refine the feature maps, *(ii)* the weights sharing concept, where a single convolutional layer is employed twice inside a specific block of layer, *(iii)* the novel multiresolution feature fusion block design used to fuse current resolution feature maps with lower/higher resolution feature maps.

ASQE-CNN operates at three patch resolutions (full, half, and quarter) and introduces in its design new network branches used to extract feature maps from the input patch and to estimate the refinement details at each resolution. ASQE-CNN is designed to operate at h×w, $\frac{h}{2}\times \frac{w}{2},$ and $\frac{h}{4}\times \frac{w}{4}$ patch resolution. In the first part of ASQE-CNN, these three resolution feature maps are obtained by processing the input patch, which lies in contrast to the general approach where the input patch is used only for processing the full-patch resolution. In the last part of ASQE-CNN, the final refinement details are extracted from the full-, half-, and quarter-patch resolutions, in contrast to the general approach where only the full-patch resolution is used.

### 3.1. Network Design

**Figure 3:**
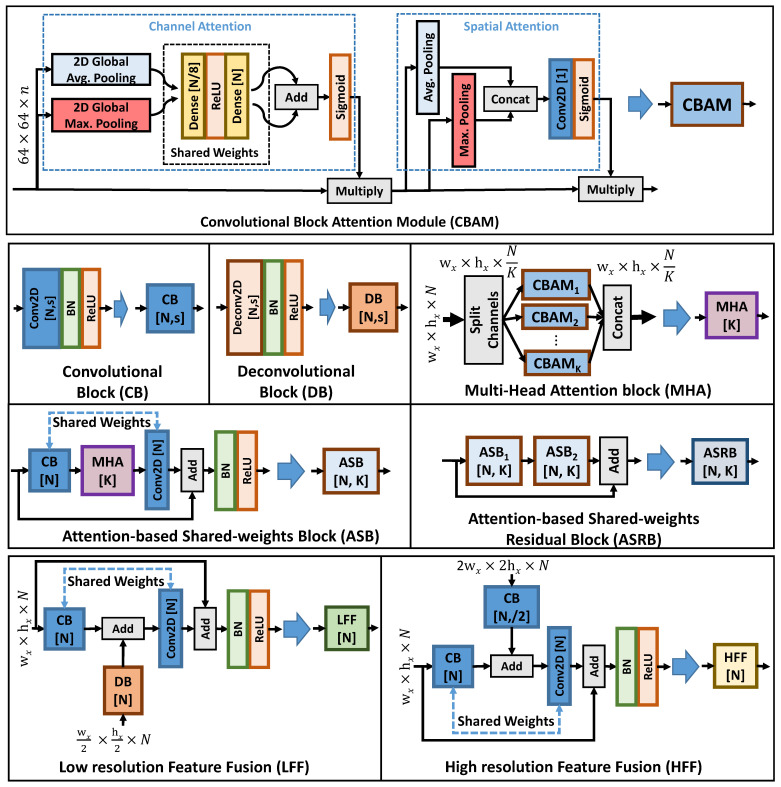
The layer structure of the eight types of blocks used to build ASQE-CNN. (**Top**) The attention mechanism implemented using the convolutional block attention module (CBAM) proposed in [44]. (**Middle**) Simple building blocks (convolutional and deconvolutional blocks) and novel attention-based blocks with weight sharing. (**Bottom**) Novel multiresolution feature fusion blocks: (**left**) low-resolution feature fusion (LFF), (**right**) high-resolution feature fusion (HFF).

The ASQE-CNN architecture is built using eight types of blocks of layers that are depicted in Figure 3.

The convolutional block attention module (CBAM) was proposed in [44] and uses both channel and spatial attention. CBAM is employed here in the design of the multi-head attention block (MHA), where the input feature maps are channel split into *K* sets of feature maps and processed separately by a different CBAM block. Hence, the MHA block is designed based on the observation that the network architectures usually contain many channels to process the current patch. Here, we propose to divide the attention into a few dozen of channels by splitting the input channels into *K* sets of feature maps.

The convolution block (CB) contains a sequence of a 2D convolution (Conv2D) layer equipped with a 3×3 kernel, *N* filters, and stride *s*; a batch normalization (BN) layer [48]; and a rectified linear unit (ReLU) activation layer [49]. Similarly, the deconvolution block (DB) contains a sequence of a deconvolution (Deconv2D) layer equipped with a 3×3 kernel, *N* filters, and stride *s*; a BN layer; and an ReLU activation layer. For simplicity, the strides s=(2,2) and s=(4,4) are denoted as “/2” and “/4”, respectively. DB is used to perform a simple block processing and to increase the patch resolution.

The attention-based shared weights block (ASB) proposes a more efficient patch processing design where an MHA block is inserted between a CB and a Conv2D layer and trainable weights are shared between the two Conv2D layers. The skip connection branch is added to the current branch after Conv2D, while BN and ReLU layers are used to further process the feature maps. Moreover, the attention-based shared weights residual block (ASRB) is introduced as a residual learning [50] bottleneck block build using ARB blocks.

The multiresolution feature fusion block design is implemented using the low-resolution feature fusion (LFF) and high-resolution feature fusion (HFF) blocks. The blocks are designed using a similar strategy as ARB, where the MHA block is replaced by an add block which combines the current feature map with the processed low/high-feature maps.

The ASQE-CNN network contains three parts. The first part is called pre-processing and is used to extract from the input patch *N* feature maps at three patch resolutions, full, half (s=/2), and quarter (s=/4), by employing three CB blocks. The second part is called multiresolution feature fusion (MFF), where the three resolution patches undergo complex processing using multiresolution feature fusion based on ARBS, HFF, and LFF blocks. To reduce the inference time, ASQE-CNN processes the full-resolution blocks using MHA blocks with K=1 and ARB blocks instead of ASRB blocks. The last part is called multiresolution refinement, where two DB blocks and three Conv2D layers, equipped with 3×3 kernels, are used to estimate the final refinement details corresponding to each resolution. One can note that ASQE-CNN adopts a new strategy for processing multiresolution patches, where the number of channels, N, remains constant throughout the network, in contrast to the general approach where the number of channels is doubled when the patch resolution is halved. Our experiments show that the increase in complexity for lower resolution patches does not provide a good improvement in network performance.

The ASQE-CNN model is obtained using N=64 and K=4, and contains around 0.89 million (M) parameters. ASQE-CNN is designed to provide an improved performance using a reduced number of parameters. Note that without applying the weight sharing approach, 1.48 M parameters must be trained. Our experiments show that consecutive Conv2D layers may contain similar weights and a too-complex architecture is affected by the vanishing gradient problem; see Section 4.6.

### 3.2. Loss Function

The loss function consists of the summation of a mean squared error (MSE)-based term and an $\ell_{2}$ regularization term, used to prevent model overfitting. Let us denote as $\Theta_{\mathrm{ASQE}-\mathrm{CNN}}$ the set of all learned parameters of the ASQE-CNN model, $X_{i}$ the *i*th input patch in the training set, and $Y_{i}$ the corresponding original patch, both of size w×h×c, where *c* denotes the number of color channels, i.e., c=3 for RGB images and c=1 for grayscale images and video sequences. Let F(·) be the function which processes $X_{i}$ using $\Theta_{\mathrm{ASQE}-\mathrm{CNN}}$ to compute the enhanced frame $\hat{Y_{i}}=F\left(X_{i},\Theta_{\mathrm{ASQE}-\mathrm{CNN}}\right)$. The loss function is formulated as follows:

(1) $$L\left(\Theta_{\mathrm{ASQE}-\mathrm{CNN}}\right)=\frac{1}{cwh}\sum_{i=1}^{cwh}∥y_{i}-{\hat{y}}_{i}{∥}_{2}^{2}+\lambda ||\Theta_{\mathrm{ASQE}-\mathrm{CNN}}{\left|\right|}_{2}^{2},$$

where $y_{i}=vec\left(Y_{i}\right)$ and $\hat{y_{i}}=vec\left(\hat{Y_{i}}\right)$ are the vectorized versions of $Y_{i}$ and $\hat{Y_{i}}$, respectively, and λ is the regularization term set here as λ=0.01. The Adam optimization algorithm [51] is employed.

## 4. Experimental Validation

The experimental setup used to validate the proposed DL-based post-filtering method is described in Section 4.1. The experimental results for quality enhancement of decoded RGB images, grayscale images, and video sequences are reported in Section 4.2, Section 4.3 and Section 4.4, respectively. The complexity of the proposed network architecture is discussed in Section 4.5. Finally, an ablation study for the proposed network design is presented in Section 4.6.

### 4.1. Experimental Setup

The paper aims to provide an efficient post-filtering method for enhancing the quality of high-resolution images and video sequences. The deep neural network models are trained using input patches extracted from the DIV2K dataset [52,53]. The training dataset in DIV2K is denoted DIV2K_train_HR and contains 800 high-resolution images. For each point reported in each rate-distortion curve, 522,240 input patches of resolution w×h=64×64 are extracted from DIV2K_train_HR. The training data is stored using 136 files, where each file contains 3840 input patches. A $\frac{125}{136}-\frac{11}{136}$ (92%−8%) ratio is used for splitting the training data into training−validation data. Each neural network model is trained during 32 epochs. The learning rate at epoch *i* is set as $\eta_{i+1}={\left(f_{d}\right)}^{\left\lfloor \frac{i}{n_{s}}\right\rfloor }\eta_{i},\forall i=1,2,\ldots ,32,$ where $f_{d}=0.2$ is the decay rate, $n_{s}=5$ is the decay step, and $\eta_{1}=5\times 10^{-4}.$

**Figure 4:**
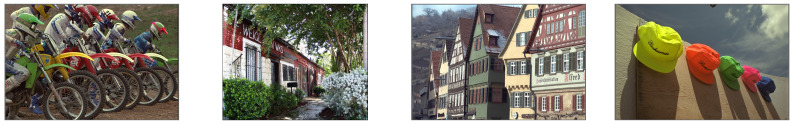
Sample images from LIVE1 [54] dataset. (Left to right) *1.bmp*, *2.bmp*, *3.bmp*, and *4.bmp*.

The experimental results for the proposed DL-based post-filtering method for quality enhancement of image and video sequences are reported on the following datasets: *(a)* *DIV2K_valid_HR*, the validation dataset from the DIV2K dataset, which contains 100 high-resolution images in the RGB color format (*801.png*, *802.png*, …, *900.png*), see Table 1; *(b)* *LIVE1* [54,55], which contains 29 images in the RGB color format, denoted here (*1.bmp*, *2.bmp*, …, *29.bmp*), see Table 1 and Figure 4.; *(c)* 15 video sequences from the Common-Test Dataset [56], denoted here by *HEVC-VTSEQ*, which contains four different video classes (see Table 2) in the YUV420 color format. In this work, the experimental validation is performed using the following three different types of input data: *(i) RGB images*, extracted from *DIV2K_valid_HR* and *LIVE1*; *(ii) grayscale images* (Y800 color format), extracted from *DIV2K_valid_HR* and *LIVE1*, where the FFmpeg [57] framework is employed to color-transform the images from RGB to YUV420 format and the grayscale images contains the luminance channel (Y); *(iii) video sequences*, from *HEVC-VTSEQ* where the results are reported only for encoding the Y channel.

The proposed method is employed to enhance the quality of both the lossy images encoded using two traditional image codecs, JPEG [1] and J2K [2], and each frame in the video sequence encoded using the previous and ongoing video coding standards, HEVC [3] and VVC [47], respectively. No modifications are applied to the codec as ASQE-CNN is employed as a post-filtering method. HEVC and VVC do apply their built-in post-filtering method. In our prior works [4,6], we proved that such a built-in method can have a negative effect on the HEVC final results if a CNN is employed.

The MATLAB implementation of the JPEG codec [1] is used to generate lossy images for each of the following six quality factors points: $q_{i}\in \left\{90,\;85,\;70,\;40,\;20,\;10\right\},$ where i=1:6. Note that the same results can be obtained using the Python Imaging Library (PIL). A neural network model is trained for each $q_{i}$ value and for each color format. Therefore, 6+6=12 neural network models are trained using DIV2K_train_HR to enhance the quality of RGB and grayscale images in DIV2K_valid_HR and LIVE1.

The MATLAB implementation of the J2K codec [2] is used to generate lossy images at the following six compression ratio points: $cr_{i}\in \left\{13,\;16,\;20,\;30,\;50,\;100\right\}.$ Similarly to JPEG, a neural network model is trained for each $cr_{i}$ value and for each color format. Hence, 12 models are trained using DIV2K_train_HR: six using RGB images and six using grayscale images. All 12 models are employed to enhance the images quality in the DIV2K_valid_HR [52] and LIVE1 [54] datasets.

The x265 library implementation of HEVC [3], available in the FFmpeg [57] framework, is employed to encode single images and video sequences using all-intra profile. For the RGB and grayscale images case, the lossy images are generated for each of the following six constant rate factors (CRF), $crf_{i}\in \left\{16,\;20,\;24,\;28,\;32,\;36\right\},$ i.e., the -c:v libx265 -preset veryslow -crf crf_i parameters are used to encode the images. A neural network model is trained for each $crf_{i}$ value and for each color format. For the video sequences case, the lossy images are generated for each of the following four standard quantization parameter (QP) values, $qp_{j}\in \left\{22,\;27,\;32,\;37\right\},$ where j=1:4, i.e., the -x265-params keyint=1:qp=qp_i parameters are used to encode the video sequences. A neural network model is trained for each $qp_{j}$ value. A total of 16 network models are trained: six models are used to enhance the grayscale images and six models to enhance the RGB images in DIV2K_valid_HR and LIVE1, and four models are used to enhance the Y component in HEVC-VTSEQ.

In this work, the VVC Test Model (VTM) [58], which is the reference software implementation of VVC [47], is employed to encode single images and video sequences in all-intra profile. Similarly to HEVC, the lossy images are generated for each of the four standard QP values. Please note that the VVC runtime is extremely large, e.g., a single high-resolution image is encoded in around 15 minutes (min), and 15(min)×4(QPvalues)×800(images)=48,000(min)≈33(days) are needed to generate training data. Four models are trained using input patches extracted from the RGB images in DIV2K_valid_HR [52] and used to report results for quality enhancement of RGB images. Four models are trained using patches extracted from the luminance channel after color transforming the images in DIV2K_valid_HR [52] and used to report results for quality enhancement of both grayscale images and video sequences. Hence, eight network models are trained: four models are used to enhance grayscale images (DIV2K_valid_HR and LIVE1) and video sequences (HEVC-VTSEQ), and four models to enhance RGB images (DIV2K_valid_HR and LIVE1).

The quality enhancement results of the proposed post-filtering method are obtained using a total of 12(JPEG)+12(J2K)+16(HEVC)+8(VVC)=48 trained network models. The results are obtained using the four tested codecs, JPEG [1], J2K [2], HEVC [3], VVC [47], and by employing the proposed method to enhance the quality of the corresponding decode image and video sequences, denoted here as JPEG+ASQE-CNN, J2K+ASQE-CNN, HEVC+ASQE-CNN, and VVC+ASQE-CNN, respectively.

In our previous work [4], a frame-wise CNN-based filtering method is proposed for video quality enhancement. In contrast to this approach, in this work, we propose a block-based approach for quality enhancement. Due to its block-based nature, the enhanced image may still be affected by blocking artifacts. The experiments show that we can further reduce the effect of coding artifacts by applying, for a second time, the proposed method on the same lossy compressed image whereby the top 16 lines and left 16 columns are cropped, i.e., the input patches are extracted a second time from the cropped input image, enhanced, and then concatenated to obtain the second enhanced image. The two enhanced images are then fused to obtain the enhancement results using alpha blending in the RGB image case and mean in the grayscale image case. Note that fusion is performed only over the size of the second enhanced image, while the top 16 lines and left 16 columns area is copied from the first enhanced image.

The image distortion is reported using both peak signal-to-noise ratio (PSNR) and structural similarity index (SSIM) [59], where the PSNR between an original image $\hat{Y}$ and a reconstructed image $\hat{Y}$ is computed as follows:

(2) $$PSNR\left[Y,\;\hat{Y}\right]=20\log_{10}\frac{255}{\sqrt{MSE[I,\;\hat{I}]}},$$

where $MSE[Y,\;\hat{Y}]$ is the corresponding mean squared error. The SSIM index [59] for *Y* and $\hat{Y}$ is computed as follows:

(3) $$SSIM\left(Y,\;\hat{Y}\right)=\frac{\left(2\mu_{Y}\mu_{\hat{Y}}+c_{1}\right)\left(2\sigma_{Y\hat{Y}}+c_{2}\right)}{\left(\mu_{Y}^{2}+\mu_{\hat{Y}}^{2}+c_{1}\right)\left(\sigma_{Y}^{2}+\sigma_{\hat{Y}}^{2}+c_{2}\right)},$$

where $\mu_{Y}$ and $\mu_{\hat{Y}}$ are the mean of *Y* and $\hat{Y}$ respectively; $\sigma_{Y}^{2}$ and $\sigma_{\hat{Y}}^{2}$ are the variance of *Y* and $\hat{Y}$ respectively; and $c_{1}={\left(k_{1}L\right)}^{2},$ $c_{2}={\left(k_{2}L\right)}^{2}$ are used to stabilize the division with weak denominator, with default values $k_{1}=0.01,\;k_{2}=0.03$, and $L=2^{b}-1$ for *b*-bit precision.

The quality enhancement results are compared using the two Bjøntegaard metrics [60], Bjøntegaard delta-rate (BD-Rate) and Bjøntegaard delta-PSNR (BD-PSNR). BD-PSNR was introduced to evaluate the quality gains made by one video codec versus another video codec at the equivalent bitrate. More exactly, when a specific amount of bits are spent to encode the video using each codec, then BD-PSNR measures by how much one codec provides a better quality than the other. Similarly, BD-rate measures the bitrate savings at the equivalent quality. In this paper, we use the Python implementation publicly available here [61]. The bitrate is measured as bits per channel (bpc), which is defined as the ratio between the compresses image file size and the product between the number of image pixels and the number of channels.

We note that in the considered experiments, we evaluate the proposed method on several data types and coding standards. The proposed method functions as a post-processing method, decoupled from the underlying coding methodology. In this sense, in contrast to customized in-loop filtering methods which require modifications of the standard implementation, the proposed method remains standard-compliant and can be used as post-filtering tool on image and video data already encoded with existing coding standards. To address the random access (RA) profile of existing video coding standards, it is expected that the proposed method will need to be performed in-loop, to maximize coding performance. Again, adapting it to in-loop filtering makes it codec-specific, and departs from the basic idea of devising a CNN-based post-processing method followed in this work. Investigating how the proposed post-filtering method can be adapted to the RA configuration is left as a topic of further investigation.

### 4.2. Quality Enhancement of RGB images

#### 4.2.1. Rate-Distortion Results

**Figure 5:**
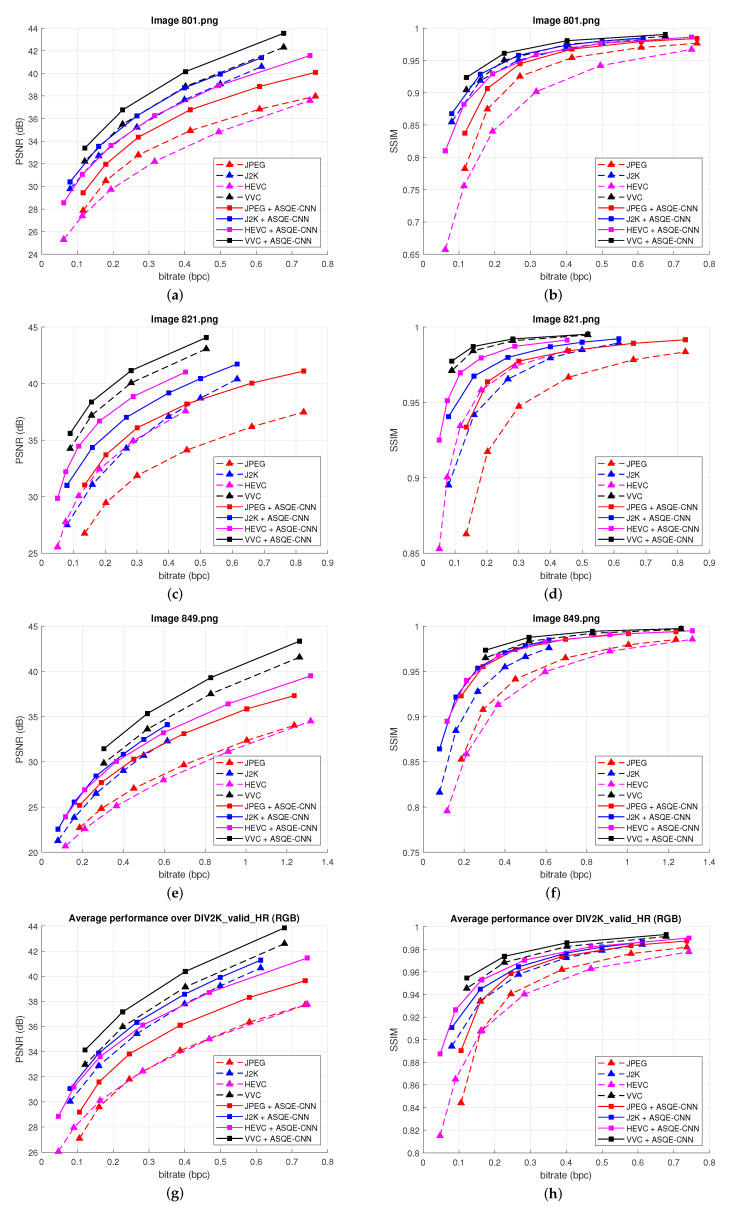
Rate-distortion results for three images, (**a**,**b**) Image *801.png*, (**c**,**d**) Image *821.png*, (**e**,**f**) Image *849.png*, and (**g**,**h**) over DIV2K_valid_HR [52]. (**Left**) bpc vs. PSNR results. (**Right**) bpc vs. SSIM results.

**Figure 6:**
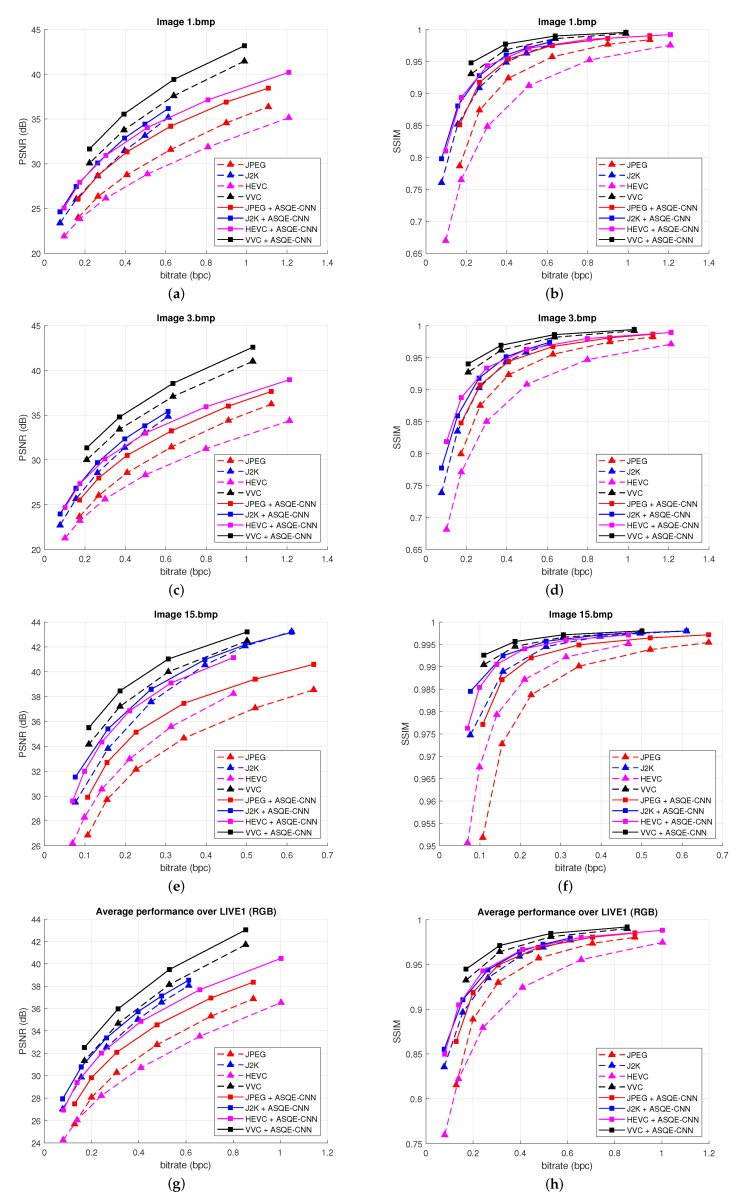
Rate-distortion results for three images, (**a**,**b**) Image *1.bmp*, (**c**,**d**) Image *3.bmp*, (**e**,**f**) Image *15.bmp*, and over LIVE1 [54], (**g**,**h**). (**Left**) bpc vs. PSNR results. (**Right**) bpc vs. SSIM results.

Figure 5 presents the rate-distortion results obtained for three randomly selected images from DIV2K_valid_HR (*801.png*, *821.png*, *849.png*) and the average results over DIV2K_valid_HR [52]. Figure 6 presents the rate-distortion results obtained for three randomly selected images from LIVE1 (*1.bmp*, *11.bmp*, *20.bmp*) and the average results over LIVE1 [54]. The quality enhancement results are measured using PSNR (left column) and SSIM (right column). One can note that the proposed method provides substantial performance improvements for both the image codecs and video coding standards. Impressive results are obtained compared with HEVC. Large BD-rate reduction is obtained compared with the newest video coding standard, VVC.

#### 4.2.2. Bjøntegaard Metrics

**Figure 7:**
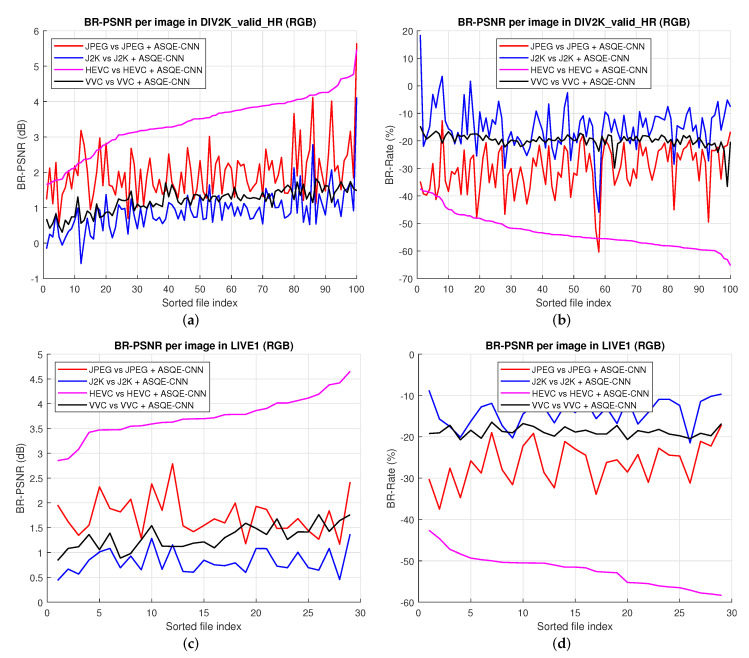
BD-PSNR results for every image in (**a**) DIV2K_valid_HR [52] and (**c**) LIVE1 [54]. BD-rate (%) results for every image in (**b**) DIV2K_valid_HR [52] and (**d**) LIVE1 [54].

Figure 7 presents quality enhancement results computed based on Bjøntegaard metrics [60] for every image in DIV2K_valid_HR [52] and LIVE1 [54]. One can note that for JPEG+ASQE-CNN, the BD-PSNR improvement varies between 0.57 dB and 5.65 dB and the BD-rate savings vary between 12.71% and 60.34%. J2K+ASQE-CNN achieves BD-PSNR improvement of up to 4.11 dB and BD-rate savings of up to 45.9%. HEVC+ASQE-CNN achieves BD-PSNR improvement of up to 5.47 dB and BD-rate savings of up to 65.26%. VVC+ASQE-CNN achieves BD-PSNR improvement of up to 1.8 dB and BD-rate savings of up to 35%.

Table 3 presents the average BD-rate savings and BD-PSNR improvement over the two test sets, DIV2K_valid_HR [52] and LIVE1 [54]. In the DIV2K_valid_HR case, ASQE-CNN provides average BD-rate savings of 31.44% and an average BD-PSNR improvement of 2.006 dB compared to the traditional JPEG codec, and average BD-rate savings of 16.02% and an average BD-PSNR improvement of 0.905 dB compared to the traditional J2K codec. However, outstanding quality enhancement results are obtained compared with the HEVC standard, average BD-rate savings of 54.61% and average BD-PSNR improvement of 3.448 dB, and compared with the VVC standard, average BD-rate savings of 19.39% and average BD-PSNR improvement of 1.201 dB. In the LIVE1 case, ASQE-CNN provides (i) average BD-rate savings of 31.44% and an average BD-PSNR improvement of 2.006 dB for JPEG; (ii) average BD-rate savings of 16.02% and an average BD-PSNR improvement of 0.905 dB for J2K; (iii) average BD-rate savings of 52.78% and an average BD-PSNR improvement of 3.7380 dB for HEVC; and (iv) average BD-rate savings of 18.79% and an average BD-PSNR improvement of 1.3056 dB for VVC. One can note that the ASQE-CNN provides impressive results over both test sets.

#### 4.2.3. Visual Results

**Figure 8:**
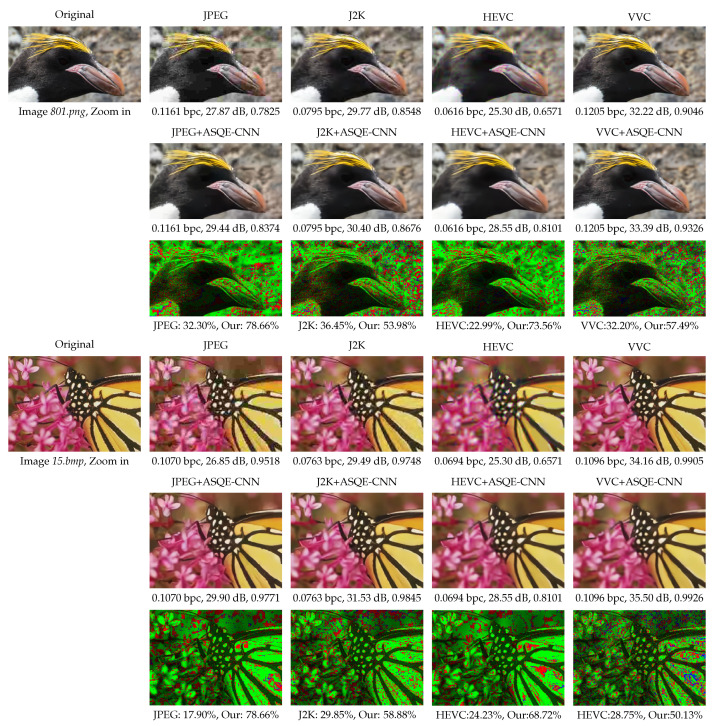
Visual results for images *801.png* from DIV2K_valid_HR [52] and *15.bmp* from LIVE1 [54]. (1st and 4th row) Original image and visual results of image and video codecs, JPEG [1], J2K [2], HEVC [3], and VVC [47]. (2nd and 5th row) Quality enhancement results corresponding to each codec. (3rd and 6th row) Pseudo-colored image comparison. Green marks the pixel positions where ASQE-CNN achieves better performance. Blue marks the pixel positions where the methods have the same performance. Red marks the pixel positions where the image/video codec (JPEG/J2K/HEVC/VVC) achieves better performance.

Figure 8 presents a visual comparison between the following methods: JPEG operating at the lowest quality parameter, $q_{6}=10$, J2K operating at the highest compression ratio, $cr_{6}=13$, HEVC operating at the highest constant rate factor, $crf_{6}=36$, VVC operating at $qp_{4}=37$, and the proposed CNN-based post-filtering method, JPEG+ASQE-CNN, J2K+ASQE-CNN, HEVC+ASQE-CNN, and VVC+ASQE-CNN, respectively. The figure shows that the quality of the highly-distorted JPEG image, having a PSNR of only 27.87 dB and SSIM of 0.7825, was substantially enhanced by JPEG+ASQE-CNN to 29.44 dB in PSNR and 0.8374 in SSIM. The coding artifacts, such as color bleeding and blocking artifacts, were reduced and most of the high details were estimated by the ASQE-CNN models, e.g., see the penguin’s beak and the rocky background. One can note that J2K offers a better compression performance than JPEG; however, in the zoom-in area the coding artifacts are still visible. Figure 8 depicts the J2K compressed image, having a PSNR of 29.77 dB and an SSIM of 0.8548, while the proposed J2K+ASQE-CNN method is able to enhance the image quality to 30.40 dB PSNR and 0.8676 SSIM, to reduce the coding artifacts and provide an improved visual quality. Moreover, in the case of HEVC compression, the image quality was improved from 25.30 dB in PSNR and 0.6571 in SSIM to 28.55 dB in PSNR and 0.8101 in SSIM, while in the case of VVC compression the image quality was improved from 32.32 dB in PSNR and 0.9046 in SSIM to 33.39 dB in PSNR and 0.9326 in SSIM.

### 4.3. Quality Enhancement of Grayscale Images

#### 4.3.1. Rate-Distortion Results

**Figure 9:**
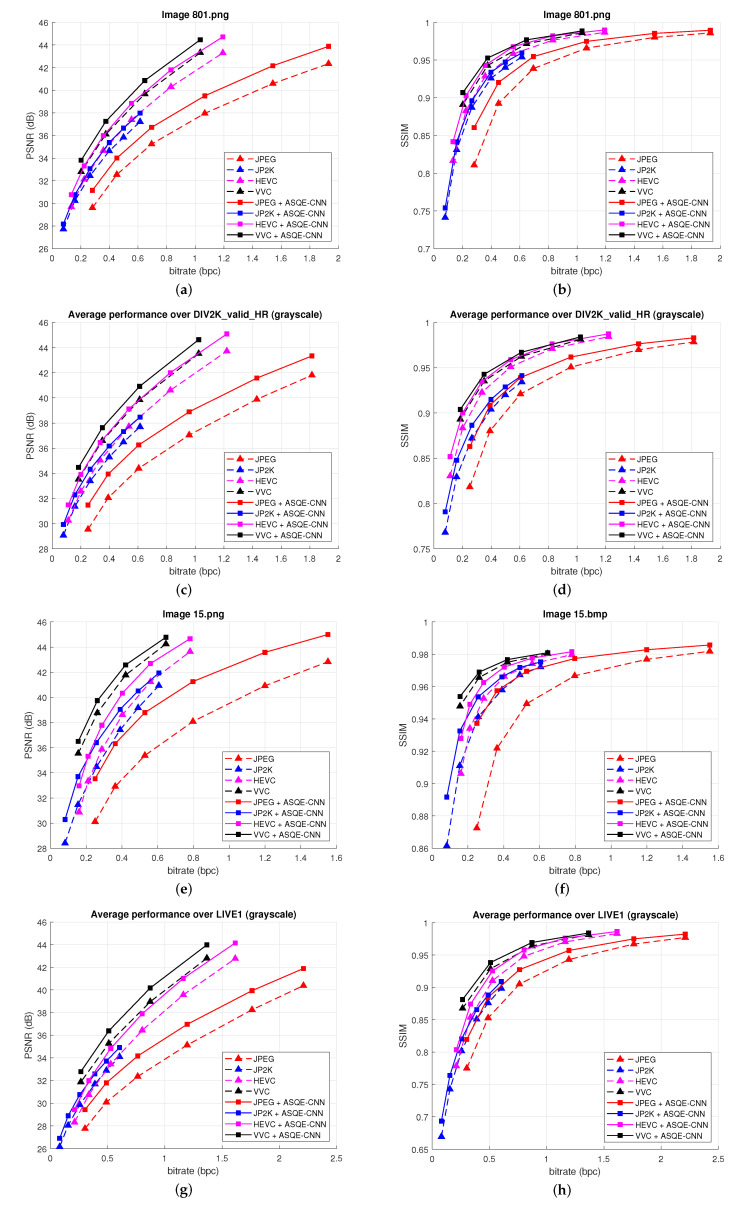
Rate-distortion results: (**a**,**b**) for Image *801.png* from DIV2K_valid_HR [52], (**c**,**d**) over DIV2K_valid_HR [52] dataset, (**e**,**f**) for Image *15.bmp* from LIVE1 [54], (**g**,**h**) over LIVE1 [54] dataset. (**Left**) bpc vs. PSNR results. (**Right**) bpc vs. SSIM results.

Figure 9 presents the rate-distortion results obtained for two images, *801.png* from DIV2K_valid_HR and *11.bmp* from LIVE1, and over the two datasets, DIV2K_valid_HR [52] and LIVE1 [54]. AEQE-CNN provides an enhanced quality for grayscale images compared to both traditional image codecs, JPEG [1] and J2K [2], and both video coding standards, HEVC [3] and VVC [47]. The image quality is improved over both tested datasets, DIV2K_valid_HR and LIVE1, having different image resolutions, which proves that the models do not overfit on the DIV2K_train_HR dataset. Moreover, impressive results are obtained compared with the most recent video coding standard, VVC [47].

Table 4 presents the comparison with state-of-the-art methods designed to enhance only lossy JPEG images: ARCNN [13], RED30 [62], DRRN [63], ARN [64], and STRNN [24]. Note that the proposed method provides the best results for all three quality factors, $q_{4}=40$, $q_{5}=20$, and $q_{6}=10$.

#### 4.3.2. Bjøntegaard Metrics

**Figure 10:**
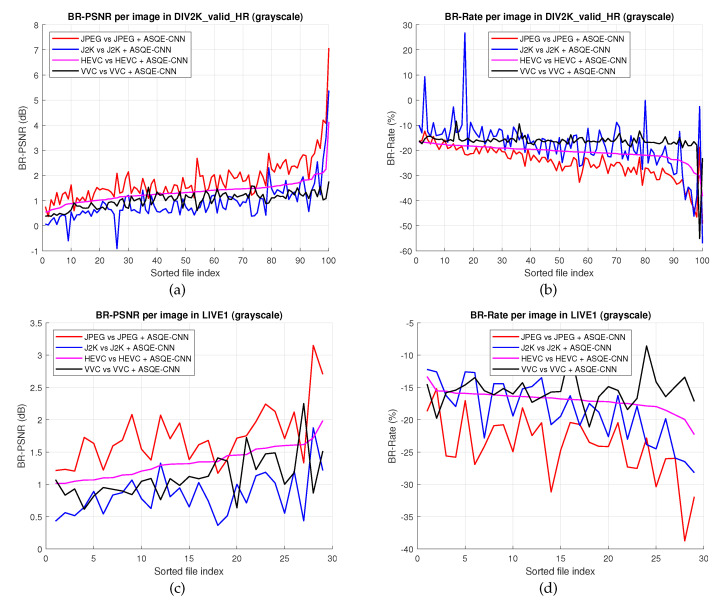
BD-PSNR results for every image in (**a**) DIV2K_valid_HR [52] and (**c**) LIVE1 [54]. BD-rate (%) results for every image in (**b**) DIV2K_valid_HR [52] and (**d**) LIVE1 [54].

Figure 10 presents quality enhancement results computed based on Bjøntegaard metrics [60] for every image in DIV2K_valid_HR [52] and LIVE1 [54]. Note that both the Y-BD-PSNR improvement and Y-BD-rate savings have a high variation over the two datasets. Table 5 presents the average BD-rate savings and Y-BD-PSNR improvement over DIV2K_valid_HR and LIVE1. In the DIV2K_valid_HR case, ASQE-CNN provides average BD-rate savings of 26.21% and an average Y-BD-PSNR improvement of 1.826 dB compared to the traditional JPEG codec, and average Y-BD-rate savings of 19.31% and an average Y-BD-PSNR improvement of 0.896 dB compared to the traditional J2K codec. Outstanding quality enhancement results are obtained compared with HEVC, average Y-BD-rate savings of 21.34% and average Y-BD-PSNR improvement of 1.267 dB, and compared with VVC, average BD-rate savings of 16.18% and average Y-BD-PSNR improvement of 1.039 dB. Similar results are obtained over the LIVE1 dataset. One can note that the ASQE-CNN provides impressive results over both test sets of grayscale images.

#### 4.3.3. Visual Results

**Figure 11:**
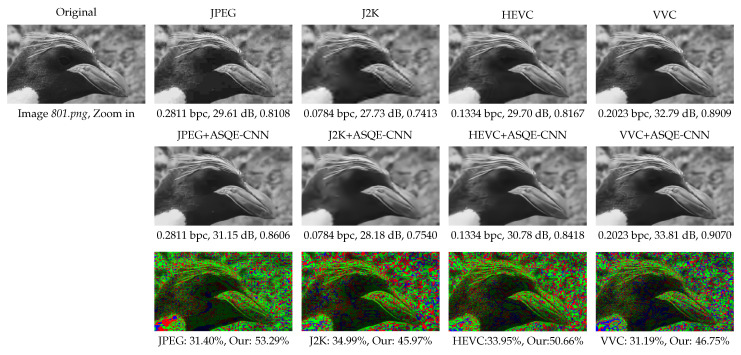
Visual results for images *801.png* in DIV2K_valid_HR [52]. (**Top**) Original image and the results of traditional codecs. (**Middle**) Quality enhancement results corresponding to each codec. (**Bottom**) Pseudo-colored image comparison. Green marks the pixel positions where ASQE-CNN achieves better performance. Blue marks the pixel positions where the methods have the same performance. Red marks the pixel positions where traditional codecs achieves better performance.

Figure 11 presents a visual comparison between JPEG ($q_{6}=10$), J2K ($cr_{6}=13$), HEVC ($crf_{6}=36$), VVC ($qp_{4}=37$), and the proposed CNN-based post-filtering method, JPEG+ASQE-CNN, J2K+ASQE-CNN, HEVC+ASQE-CNN, and VVC+ASQE-CNN, respectively. The quality of the traditional methods is substantially enhanced by ASQE-CNN. The coding artifacts are reduced and most of the high details are estimated by the ASQE-CNN models, e.g., see the penguin’s beak and the rocky background. The bottom row presents the pseudo-color image compression between the above two image quality results, where green marks the pixel positions where the proposed ASQE-CNN method provides an improved performance, and red marks the pixel positions where it fails. The proposed method estimates fine details for around 50% of the pixels and provides the same result for around 20% of the pixels.

### 4.4. Quality Enhancement of Video Sequences

#### 4.4.1. Bjøntegaard Metrics

Table 6 presents the video quality enhancement results computed as Y-BD-rate savings for HEVC-vTEQ [56]. The results are computed for all available frames in each video sequence by employing ASQE-CNN to post-process the HEVC [3]-decoded videos and are compared with the results of the six most recent state-of-the-art methods, VR-CNN [15], FE-CNN [26], MLSDRN [37], RRCNN [28], VRCNN-BN [18], and FQE-CNN [4]. The results obtained for the first 30 frames in each video sequence by employing ASQE-CNN to post-process the VVC [47]-decoded videos are also reported in Table 6. ASQE-CNN achieves an outstanding performance compared with the state-of-the-art methods for all video sequences, except for B5, and provides 15.47% Y-BD-rate savings compared with HEVC and 14.66% Y-BD-rate savings compared with VVC.

Table 7 presents quality enhancement results computed as Y-BD-PSNR improvement. ASQE-CNN provides an improvement of around 1.13 dB compared with HEVC [3] and 0.88 dB compared with VVC [47].

#### 4.4.2. Visual Results

**Figure 12:**
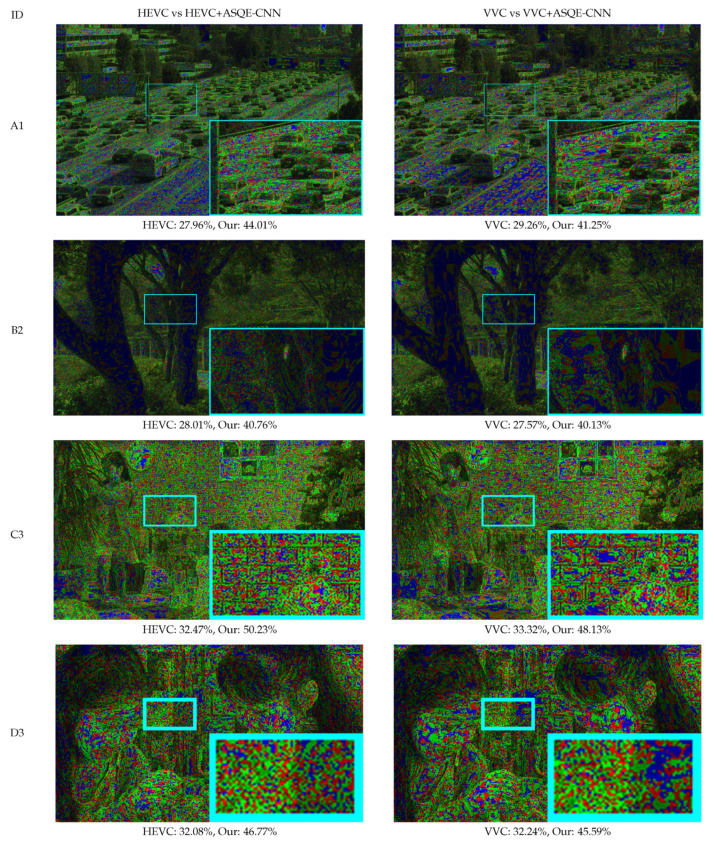
Pseudo-colored image comparison. Green marks the pixel positions where ASQE-CNN achieves better performance. Blue marks the pixel positions where the methods have the same performance. Red marks the pixel positions where HEVC/VVC achieves better performance. The bottom-right corner of each image presents the zoom in of the RoI marked by the cyan rectangle.

Figure 12 presents the pseudo-colored image comparison between HEVC and VVC at $qp_{4}=37$ and HEVC+ASQE-CNN, and VVC+ASQE-CNN, respectively. ASQE-CNN estimates fine details for ≈45% of the pixels and provides the same result for ≈25% of the pixels.

### 4.5. Complexity

ASQE-CNN is designed to have a medium complexity and small inference time. It is implemented in the *Python* programming language using the *Keras* [65] open-source deep learning library, and is run on machines equipped with NVIDIA TITAN Xp GPU. Each model is trained in around 34 h (1.42 days). The average inference time for a batch of 32 input patches is 67 ms. For example, for filtering a lossy compressed image with a 1356×2040 resolution, 704 input patches are extracted and an inference time of around $\frac{704}{32}\cdot 67=1474$ ms is required to enhance the lossy compressed image.

### 4.6. Ablation Study

The ASQE-CNN architecture is designed based on the following three main concepts: (a) the attention mechanism; (b) the weight sharing approach; and (c) the proposed MFF modules. Here, we study how important is to integrate each one of these concepts in ASQE-CNN. The first version is designed without the use of the attention mechanism, where all the MHA blocks are removed. This version is called noAttention. The second version is designed without the use of the weight sharing approach. Therefore, the ASB and LFF and HFF blocks are designed using two convolutional layers with sparely trained weights instead of a single convolutional layer employed twice inside the block. This version is called noWeighSharing. The third version is designed without the use of the MFF modules, i.e., by following the classical multiresolution patch processing design (U-Net). In this case, the following branches in the proposed ASQE-CNN architecture are removed: *(1)* the half- and quarter-resolution branches which extract feature maps from the input patch in the first part of the architecture; and *(2)* the half- and quarter-resolution branches which provide the extra refinement in the last part of the architecture. This version is called noMFF (or U-Net).

**Figure 13:**
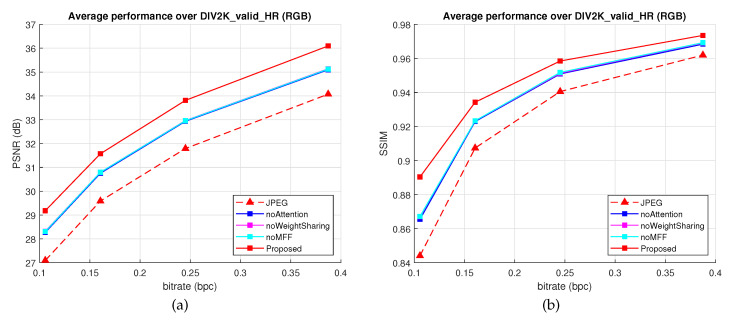
Ablation study of the ASQE-CNN architecture. Average performance over the DIV2K_valid_HR [52] dataset: (**a**) bpc-vs-PSNR results; (**b**) bpc vs. SSIM results.

Figure 13 presents the average performance results over DIV2K_valid_HR [52], while Table 8 presents the average Bjøntegaard metrics and the runtime using batch size (bs) of 100 input patches. All three versions are affected by a performance drop compared with the proposed architecture. The following conclusions can be drawn: (1) the attention mechanism has the highest influence in the network design of around 12% BD-rate savings compared with the proposed architecture; (2) although noWeighSharing is more complex (contains more parameters), it provides a small improvement of around 11.45% BD-rate savings compared with the proposed architecture; (3) the classical multiresolution patch processing design (U-Net) was improved using the proposed MFF modules, which provides around 11.54% BD-rate savings compared with the proposed architecture.

## 5. Conclusions

The paper introduces a novel CNN-based post-filtering method for enhancing the quality of lossy images encoded using traditional codecs and video sequences encoded using recent video coding standards. A novel deep neural network architecture is proposed to estimate the refinement details. ASQE-CNN incorporates in its design three concepts: the attention mechanism implemented using a multi-head attention block, the weights sharing concept, where a single convolutional layer is employed twice in a specific block, and the proposed MFF blocks, LFF and HFF, designed to fuse current resolution feature maps with lower/higher-resolution feature maps. It operates at three resolutions and uses new network branches to extract feature maps from the input patch and to estimate refinement details at each resolution. Experimental results demonstrate substantial average BD-rate and BD-PSNR improvements over traditional image and video codecs. The paper demonstrates the potential of CNN-based post-filtering methods for widely-used codecs.

## Figures and Tables

**Table 1:** Image datasets.

Usage	Dataset Name	Number of Images	Megapixels
Training	DIV2K_train_HR [52]	800	1.3–4 MP
Testing	DIV2K_valid_HR [52]	100	1.6–4 MP
Testing	LIVE1 [54]	29	0.28–0.39 MP

**Table 2:** HEVC-VTSEQ: the Common-Test Dataset [56].

ID	Video Sequence Name	Number of Frames	Class	Frame Resolution
A1	*Traffic*	150	A	2560×1600
A2	*PeopleOnStreet*	150		
B1	*Kimono*	240	B	1920×1080
B2	*ParkScene*	240		
B3	*Cactus*	500		
B4	*BQTerrace*	600		
B5	*BasketballDrive*	500		
C1	*RaceHorses*	300	C	832×480
C2	*BQMall*	600		
C3	*PartyScene*	500		
C4	*BasketballDrill*	500		
D1	*RaceHorses*	300	D	416×240
D2	*BQSquare*	600		
D3	*BlowingBubbles*	500		
D4	*BasketballPass*	500		

**Table 3:** Average Bjøntegaard metrics for RGB images.

Method	BD-Rate (%)		BD-PSNR (dB)	
	DIV2K [52]	LIVE1 [54]	DIV2K [52]	LIVE1 [54]
JPEG+ASQE-CNN	−31.44	−26.59	2.006	1.7442
J2K+ASQE-CNN	−16.02	−14.26	0.905	0.8190
HEVC+ASQE-CNN	−54.61	−52.78	3.448	3.7380
VVC+ASQE-CNN	−19.39	−18.79	1.201	1.3056

**Table 4:** Average PSNR (dB) results over LIVE1 [54] for grayscale images.

$q_{i}$	JPEG [1]	ARCNN [13]	RED30 [62]	DRRN [63]	ARN [64]	STRNN [24]	JPEG+ ASQE-CNN
10	27.77	28.73	29.32	29.21	29.27	29.38	**29.42**
20	30.07	30.89	31.69	31.19	31.34	31.72	**31.79**
40	32.35	33.63	−	−	33.13	34.06	**34.16**

**Table 5:** Average Bjøntegaard metrics for grayscale images.

Method	Y-BD-Rate (%)		Y-BD-PSNR (dB)	
	DIV2K [52]	LIVE1 [54]	DIV2K [52]	LIVE1 [54]
JPEG+ASQE-CNN	−26.21	−24.29	1.826	1.7415
J2K+ASQE-CNN	−19.31	−19.23	0.896	0.8436
HEVC+ASQE-CNN	−21.34	−17.32	1.367	1.3539
VVC+ASQE-CNN	−16.18	−15.28	1.039	1.1171

**Table 6:** Video quality enhancement results computed as Y-BD-rate (%) savings for HEVC-vTSEQ.

ID	HEVC [3] Codec							VVC [47]
	(All Frames)							(30 Frames)
	VR-CNN	FE-CNN	MLSDRN	RRCNN	VRCNN-BN	FQE-CNN	Proposed	
	[15]	[26]	[37]	[28]	[18]	[4]	ASQE-CNN	
A1	−5.6	−6.6	−	−9.8	−8.7	−11.0	**−18.46**	−14.04
A2	−5.4	−5.8	−	−8.7	−9.3	−9.6	**−17.09**	−11.85
B1	−2.5	−5.7	−6.2	−6.6	−11.8	−8.4	**−19.02**	−16.32
B2	−4.4	−5.1	−6.5	−7.6	−8.7	−8.0	**−18.12**	−20.43
B3	−4.6	−4.9	−5.8	−7.0	−10.5	−7.7	**−14.10**	−15.46
B4	−2.6	−2.5	−3.7	−3.3	−7.7	−4.3	**−12.87**	−12.39
B5	−2.5	−5.2	−4.4	−6.7	**−14.5**	−8.5	−13.37	−14.35
C1	−4.2	−4.2	−4.7	−6.3	−6.7	−8.4	**−14.65**	−14.59
C2	−5.1	−5.4	−6.0	−9.9	−7.5	−11.8	**−15.94**	−15.87
C3	−3.6	−3.6	−4.3	−5.9	−4.0	−6.5	**−13.43**	−14.45
C4	−6.9	−8.6	−8.3	−15.1	−8.5	−14.7	**−19.54**	−16.34
D1	−7.6	−7.7	−8.9	−10.0	−6.6	−9.6	**−14.20**	−14.93
D2	−3.8	−4.2	−4.3	−7.4	−3.4	−6.8	**−11.61**	−12.82
D3	−4.9	−5.0	−5.7	−6.8	−4.3	−7.0	**−12.32**	−15.05
D4	−5.3	−5.9	−6.6	−10.0	−7.4	−10.6	**−12.74**	−15.55
A	−5.5	−6.2	−	−9.3	−9.0	−10.3	**−17.77**	−12.95
B	−3.3	−4.7	−5.3	−6.2	−10.6	−7.4	**−15.51**	−15.79
C	−5.0	−5.5	−5.8	−9.3	−6.7	−10.4	**−15.89**	−15.31
D	−5.4	−5.7	−6.4	−8.6	−5.4	−8.5	**−12.72**	−14.59
All	−4.8	−5.5	−	−8.4	−8.3	−9.2	**−15.47**	−14.66

**Table 7:** Video quality enhancement results computed as Y-BD-PSNR improvement over HEVC-vTSEQ [56].

Class	HEVC [3]	VVC [47]
(Average)	(All Frames)	(30 Frames)
A	1.2007 dB	0.7551 dB
B	0.7827 dB	0.6743 dB
C	1.2006 dB	1.0315 dB
D	1.3279 dB	1.0689 dB
ALL	1.1280 dB	0.8824 dB

**Table 8:** Average Bjøntegaard metrics for RGB images.

Method	BD-Rate	BD-PSNR	#param	Time (bs = 100)
noAttention	−19.51%	1.1400 dB	0.83 M	144 ms
noWeightSharing	−20.10%	1.1772 dB	1.48 M	169 ms
noMFF (U-Net)	−20.01%	1.1701 dB	0.56 M	113 ms
Proposed	−31.55%	2.0166 dB	0.89 M	165 ms

## Abbreviations

The following abbreviations are used in this manuscript:

JPEG	Joint Photographic Experts Group
HEVC	High-efficiency video coding
VVC	Versatile video coding
CNN	Convolutional neural network
MPEG	Moving Picture Experts Group
ASQE-CNN	Attention-based shared weights quality enhancement CNN
CB	Convolutional block
DB	Deconvolutional block
CBAM	Covolutional block attention module
ASB	Attention-based shared weights block
ASRB	Attention-based shared weights residual block
MHA	Multi-head attention block
LFF	Low-resolution feature fusion
HFF	High-resolution feature fusion
